# High interferon type I responses in the lung, plasma and spleen during highly pathogenic H5N1 infection of chicken

**DOI:** 10.1186/1297-9716-42-6

**Published:** 2011-01-11

**Authors:** Hervé R Moulin, Matthias Liniger, Sylvie Python, Laurence Guzylack-Piriou, Manuela Ocaña-Macchi, Nicolas Ruggli, Artur Summerfield

**Affiliations:** 1 Institute of Virology and Immunoprophylaxis (IVI), Sensemattstrasse 293, 3147 Mittelhäusern, Switzerland; 2 Agroscope Changins-Wädenswil (ACW), Route de Duillier, 1260 Nyon, Switzerland; 3 INRA, Unité de Pharmacologie-Toxicologie, 180 chemin de Tournefeuille, 31931 Toulouse, France

## Abstract

This study shows that high pathogenic H5N1 influenza virus infection of chicken induced high levels of bioactive interferon type I in the lung (4.3 × 10^5 ^U/mg tissue), plasma (1.1 × 10^5 ^U/mL), and spleen (9.1 × 10^5 ^U/mg tissue). In contrast, a low pathogenic attenuated H5N1 vaccine strain only induced approximately 24 times less IFN in the lung, 441 times less in the spleen and 649 less in the plasma. This was in the same range as a reassortant carrying the HA from the vaccine strain and the remaining genes from the high pathogenic virus. On the other hand, a reassortant virus with the HA from the high pathogenic H5N1 with the remaining genes from the vaccine strain had intermediate levels of IFN. The level of interferon responses related to the viral load, and those in the spleen and blood to the spread of virus to lymphoid tissue, as well as disease severity. In vitro, the viruses did not induce interferon in chicken embryonic fibroblasts, but high levels in splenocytes, with not clear relationship to pathogenicity and virulence. This, and the responses also with inactivated viruses imply the presence of plasmacytoid dendritic cell-like leukocytes within the chicken immune system, possibly responsible for the high interferon responses during H5N1 infection. Our data also indicate that the viral load as well as the cleavability of the HA enabling systemic spread of the virus are two major factors controlling systemic IFN responses in chicken.

## Introduction

Although avian influenza virus (AIV) has been shown to inhibit interferon type I (IFN) induction by its NS1 protein, large amounts of IFN can be detected upon infection of mammals [1-7]. The observed correlation of IFN in respiratory secretions to symptom severity and viral load [5,8] indicate a possible role in disease pathogenesis. Mouse models show that the major source of this IFN are plasmacytoid dendritic cells (pDC) [9], representing a specialized cell type for the production of IFN [10]. pDC, in contrast to epithelial cells or macrophages are not inhibited by the action of NS1 [11]. On the other side, AIV with defective NS1 are strongly attenuated in mice [3] and in chicken [12-15] probably related to IFN induction in epithelial cells, contrasting to AIV with functional NS1 [13-15].

Despite this information, the contribution of IFN to disease and protection in chicken is unclear. Furthermore, no information is available whether the chicken immune system does possess pDC-like cells, which would be able to mount strong IFN responses against AIV with functional NS1. AIV can be divided into highly and low pathogenic AIV pathotypes (HPAIV and LPAIV, respectively). While LPAIV mainly replicate in the respiratory and digestive tract of chicken, HPAIV possess a polybasic hemagglutinin (HA) cleavage site sensitive to ubiquitous proteases, providing the virus the capacity to replicate in multiple organs including lymphoid tissue, and thereby causing severe disease and high mortality [16,17]. Considering that the frequency of pDC is elevated in lymphoid tissue in mammals and assuming that chicken also possess a analogous cellular system, it could be speculated that such differences in tropism should have a major impact on systemic IFN responses. In one study, after lethal H5N1 infection of chicken, no IFN-α or IFN-β mRNA was detected in the lung, spleen or caecal tonsils although high levels of the IFN-inducible genes Mx and PKR were found [18]. In contrast, others found IFN-α transcripts in lungs of H5N1 infected chicken, but only transiently [19]. Consequently, the main aim of this study was to investigate H5N1-induced local and systemic IFN bioactivity, avoiding the problem caused by the short half-life of IFN mRNA. Furthermore, we investigated whether chicken leukocytes can produce IFN in response to influenza virus.

## Materials and methods

### Viruses

We employed a HPAIV H5N1 virus (A/chicken/Yamaguchi/7/04, [20], "Yama", GenBank AB166859 to AB166866) and a LPAIV H5N1 vaccine strain (A/vac-1/Hokkaido/04, [21], "Vac", GenBank AB259709 to AB259716) as well as two reassortant viruses by exchanging the HA in both directions using reverse genetic as previously described [22]. These viruses were termed Yama-V/HA (Yama with Vac HA, GenBank AB259712.1) and Vac-Y/HA (Vac with Yama HA, AB166862.1). The Yama and Vac HA amino acid sequences were 93% identical. Before use, the viruses were passaged once in embryonated chicken eggs. In addition, with used the following natural isolates, all kindly provided by Dr. William Dundon (IZSV, Italy): H7N3 A/Turkey/Italy/4616/03 (LPAIV), H5N2 A/Turkey/Italy/1258/05 (LPAIV), H7N1 A/Turkey/Italy/3675/99 (LPAIV), H5N1 A/Turkey/Turkey/05 (HPAIV), H7N1 A/Turkey/Italy/4580/99 (HPAIV), all grown in embryonated chicken eggs. Inactivation of the viruses employed 2-bromoethylamine hydrobromide (BEI) treatment as previously described [22]. Titers were determined by end-point titrations on MDCK cells [22].

### Cell culture, splenocytes isolation and stimulation

Madin-Darby canine kidney (MDCK) cells were propagated in MEM (Invitrogen Basel, Switzerland) supplemented with 10% fetal bovine serum (FBS; Biowest Nuaillé France), non-essential amino acids (Invitrogen, Basel, Switzerland) and 1 mM natrium pyruvate (Invitrogen). Human embryonic kidney (HEK) 293T cells were propagated in DMEM GlutaMax (Invitrogen) supplemented with 10% FBS. Chicken embryo fibroblasts (CEF) were prepared from 10-day-old embryonated chicken eggs by trypsin digestion and cultured in DMEM GlutaMax containing 7% chicken serum. Chicken splenocytes were isolated by cutting spleen into small pieces, passing the cells through a 40 μm cell strainer (Becton Dickinson, Basel Switzerland), and then purified by Ficoll gradient centrifugation (1.077 g/mL, GE Healthcare, Glattbrugg, Switzerland; 600 × *g*, 20 min). After washing twice with PBS (250 × *g*, 10 min), the cells were resuspended in DMEM, 10% FBS and 2% autologous chicken serum in 96-well-plates at 2.5 × 10^5 ^cells/well, and immediately stimulated with the viruses as indicated for 24 h.

### Bioassay for IFN type I

IFN was detected with a bioassay using CEC-32/Mx-Luc cells expressing luciferase under the control of the Mx promoter. Confluent monolayers were incubated with the samples for 6 h as previously described. Recombinant chicken IFN-α was used as standard [23]. All samples were heat-treated for 30 min at 65°C to destroy virus infectivity. This treatment also destroyed any IFN-inducing ability of influenza virus in various cell culture systems, without affecting the activity of recombinant chicken IFN-α (data not shown). Both CEC-32/Mx-Luc cells and chicken IFN-α were kindly obtained from Dr Peter Staeheli (University of Freiburg, Germany).

### Animal experiment

A total of 32 6-weak old specific-pathogen-free leghorn chicken were infected with the Yama, Vac, Yama-V/HA and Vac-Y/HA viruses at 10^6 ^TCID_50 _diluted in 0.5 mL PBS intratrachealy (8 animals per group) using a blunt-ended cannula. Three animals inoculated with PBS served as controls. Four animals from each group were killed after 24 h to determine early IFN responses in the lungs, plasma, and spleen. The remaining animals were kept for clinical examination and collection of tracheal and cloacal swabs. Animals in a moribund state were euthanized. The animal experiment was approved by the local animal welfare committee of the Canton of Berne, Switzerland.

### Viral RNA isolation and quantification

RNA was extracted from swabs using a kit from Macheray-Nagel and amplified by real-time RT-PCR using primers and probe as described [24,25]. For RNA isolation of lung and spleen, 1 g was homogenized in Trizol (Invitrogen) with a Ultra-Turrax (Sigma), and RNA was isolated with the Trizol method as described by the manufacturer. This was also employed for the plasma samples. Quantitative RT-PCR was carried out with a QuantiTect Probe RT-PCR kit (Qiagen, Hombrechtikon, Switzerland) on a 7500 Fast Real-Time PCR system (Applied Biosystems, Rotkreuz, Switzerland). Mean Ct values were determined from triplicates. For absolute RNA quantification, an internal standard was used based on M1 genome copies.

## Results

### HPAIV and LPAIV induce IFN type I in chicken splenocytes

**Figure 1:**
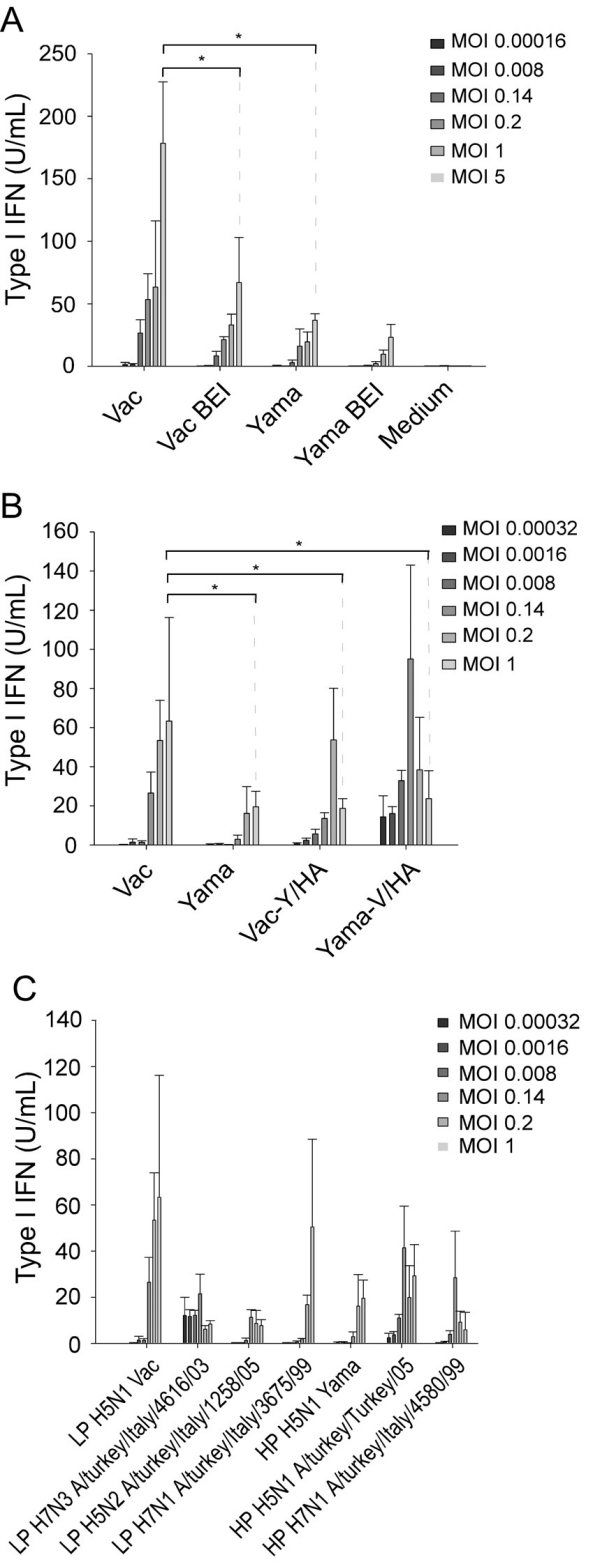
**H5N1 AIV induce IFN in chicken splenocytes**. Splenocytes were stimulated with various doses of AIV for 24 h. In (A), live and BEI-inactivated Vac and Yama viruses are shown. In (B), Vac and Yama are compared with the reassortants Vac-Y/HA and Yama-V/HA. In (C), various natural isolates of LPAIV and HPAIV are shown. IFN was quantified by bioassay. The averages of triplicate cultures with standard deviations are shown. The * indicates statistical differences calculated with the students *t*-test (p < 0.05). The results are representative for at least three independent experiments.

Relating to the known function of NS1 as IFN antagonist [13,14], none of the viruses employed (all listed in Materials and methods) was able to induce bioactive IFN in chicken embryo fibroblasts or DF-1 at doses of 0.4, 2 and 10 TCID_50_/cell (data not shown). This contrasted with cell cultures of chicken splenocytes. Both, HPAIV and LPAIV H5N1 viruses induced IFN in a virus dose-dependent manner, with Vac triggering higher responses but only at the highest m.o.i. of 5 TCID_50_/cell (Figure 1A). Considering that in mammals, plasmacytoid dendritic cells (pDC) are the source of type I IFN within the leukocyte compartment after influenza virus stimulation [10], we next investigated the effect of virus inactivation on splenocyte responses to evaluate the possibility that in chicken a similar cell type would exist. Inactivation of the viruses with BEI did not significantly reduce IFN responses except for the highest of the Vac virus (Figure 1A).

Considering the potential differences found between HPAIV and LPAIV we next investigated the role of the HA in IFN response of splenocytes. Our results demonstrate that introducing the HA of Vac into the Yama enhanced its IFN inducing activity, but Vac-Y/HA did not clearly differ to Yama (Figure 1B). Furthermore, when a number of other HPAIV and LPAIV isolates were tested for their capacity to induce IFN, no clear relationship between IFN induction and pathogenicity of the AIV was found (Figure 1C). With some of the virus preparations lower responses were observed at high virus doses probably reflecting unspecific toxicity present in some of CAF preparations.

### Infection of chicken: survival and virus excretion

**Figure 2:**
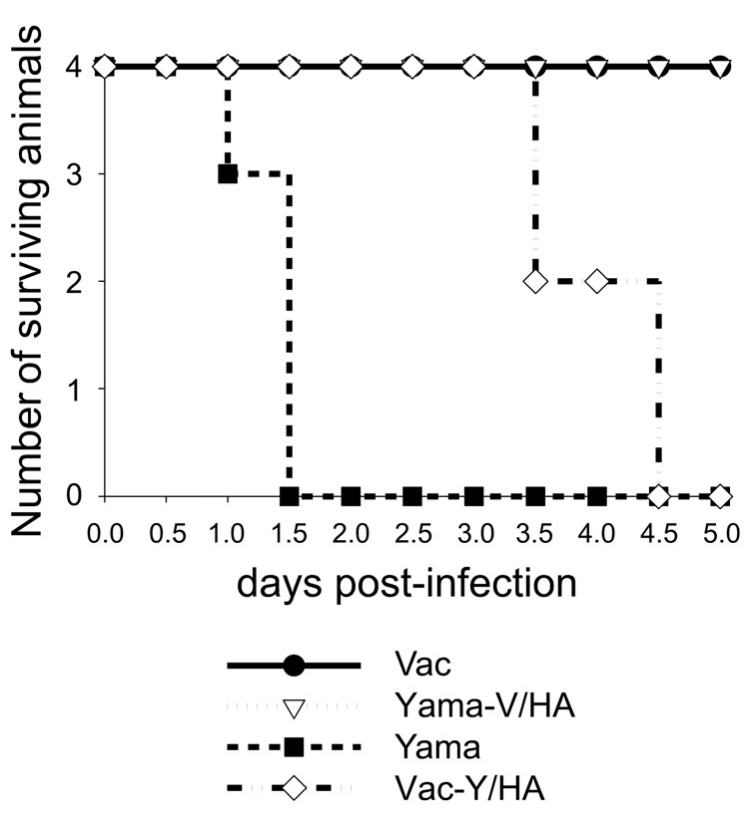
**Survival of chicken following infection with H5N1 Vac, Yama, Vac-Y/HA and Yama-Vac/HA**. Animals in moribund state were euthanized.

Figure 2 demonstrates that the Yama virus induced a peracute disease with 100% mortality within 36 h, corresponding to previous reports [19]. The Vac-Y/HA was also highly virulent with 100% mortality but with a 48 h delay. Before death, these animals showed severe depression starting at two days post infection. In contrast, no disease was observed with Vac and Yama-V/HA, the latter only causing a mild depression at 24-48 h.

As shown in Table 1, only one animal of the Vac group had weakly positive tracheal swabs at 24 h post infection (pi), but all Yama-V/HA infected animals were clearly positive at 2 days pi. The high quantities of viral RNA detected in the swabs of the Yama and Vac-Y/HA virus-infected birds related to their disease severity.

**Table 1:** Quantification of viral RNA in tracheal and cloacal swabs

		1 dpi				2 dpi				3 dpi				4 dpi				5 dpi			
		
		#1	#2	#3	#4	1	#2	#3	#4	#1	#2	#3	#4	#1	#2	#3	#4	#1	#2	#3	#4
Vac	Trachea	-	-	-	-	-	-	-	±	-	-	-	-	-	-	-	-	-	-	-	-
	Cloaca	-	-	-	-	-	-	-	±	-	-	-	-	-	-	-	-	-	-	-	-
Yama-V/HA	Trachea	-	-	-	+	+	+	+	+	-	-	-	+	-	-	-	-	-	-	-	-
	Cloaca	-	-	-	-	-	-	-	-	-	-	-	-	-	-	-	-	-	-	-	-
Yama	Trachea	++	++	++	++		†	†	†	†	†	†	†	†	†	†	†	†	†	†	†
	Cloaca	++	++	++	++	†	†	†	†	†	†	†	†	†	†	†	†	†	†	†	†
Vac-Y/HA	Trachea	+	-	-	-	+	+	+	+	+	±	+	+	+	+	†	†	†	†	†	†
	Cloaca	-	-	-	-	±	-	+	-	+	++	++	-	++	+	+++	+++	†	†	†	†

- PCR negative; ± 45-Ct < 5; + 45-Ct 5-15; ++ 45-Ct 25-15; +++ 45-Ct > 25

† animal dead

### Viral RNA and IFN quantities in lung, blood and spleen

To investigate the relationship of viral load to IFN in the lung, blood and spleen, four chickens per group were sacrificed at 24 h pi and viral RNA was isolated from lung and spleen and subjected to real-time RT-PCR.

**Figure 3:**
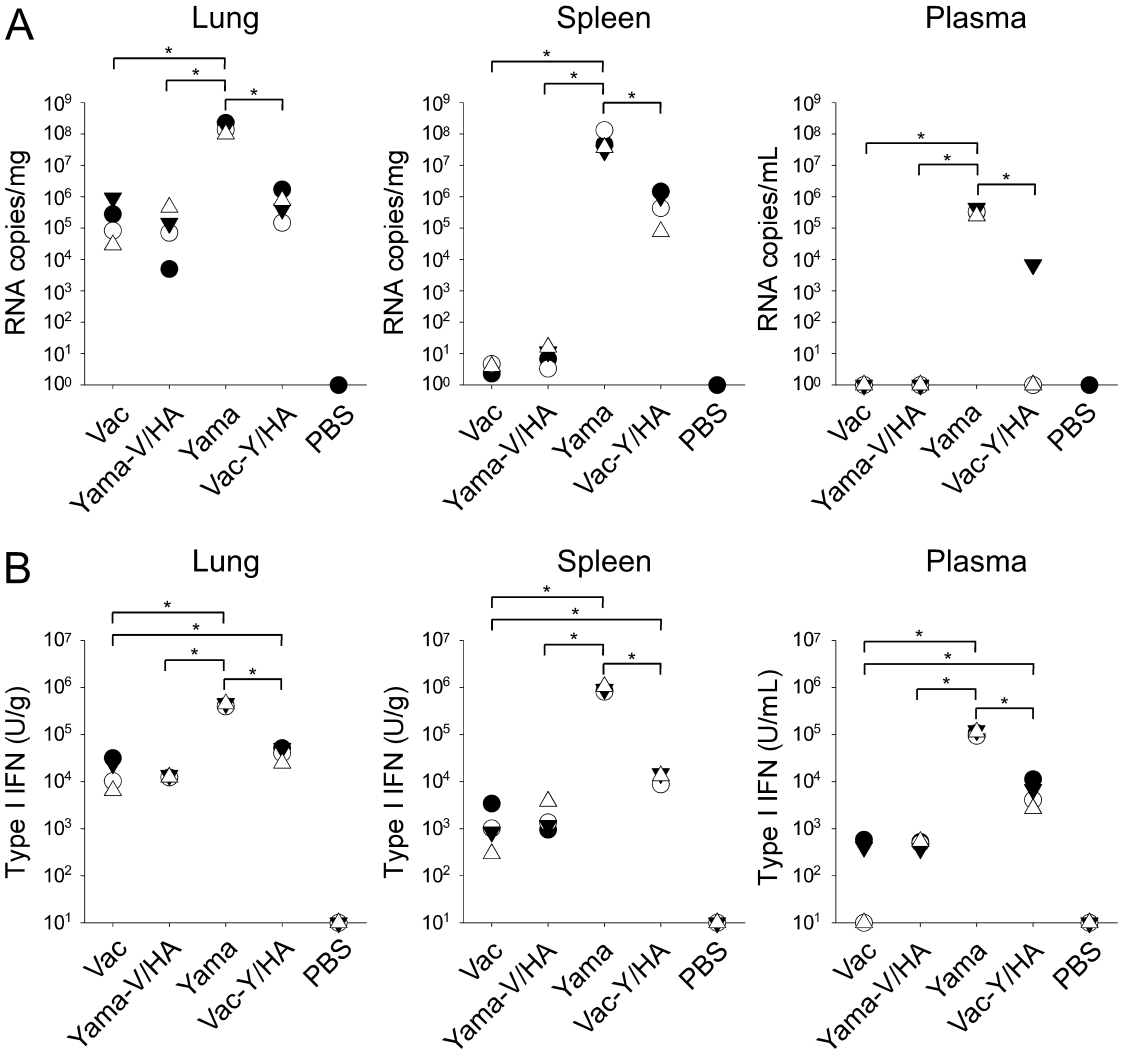
**Viral load and IFN in lung, spleen and plasma of Vac, Vac-Y/HA, Yama and Yama-V/HA at 24 h pi**. PBS-inoculated animals were used as controls (PBS). (A.) Copy numbers of M1 RNA quantified by real-time RT-PCR. (B.) IFN levels quantified by bioassay. Each symbol represents a different animal. The * indicates statistical differences calculated with the Mann-Whitney Rank Sum Test (p < 0.05).

Figure 3A demonstrates that viral RNA loads in the lung of animals infected with Vac, Yama-V/HA and Vac-Y/HA viruses were similar, contrasting with a 10^3^-10^4^-fold increase found with the Yama virus. This related to the IFN found in the lung, with high levels in the lung of all infected chicken, but 10-50 times higher levels after Yama virus infection (Figure 3B).

As expected only viruses with polybasic HA cleavage site were able to spread efficiently to the spleen. While only 1-20 M1 copies/mg spleen were detected after infection with Vac and Yama-V/HA, 10^5^-10^6 ^copies were found with Vac-Y/HA and even 10^7^-10^8 ^with Yama (Figure 3A). Again, this related to the levels of IFN found in this organ. While all viruses induced IFN, the levels were approximately 10^2^-10^3 ^times higher with Yama when compared to Vac and Yama-V/HA. IFN in the spleen of Vac-Y/HA virus-infected birds was around 10 times over those induced by Vac.

No virus was detected in the plasma of animals infected with LPAIV and also three of the animals infected with Vac-Y/HA. In contrast, high viral loads were found after Yama virus infection. Despite the lack of detectable virus in the LPAIV infected chicken, plasma IFN was detectable in most animals, but Yama virus induced 10^3^-10^4^-fold higher levels (Figure 3B).

## Discussion

This study demonstrates that infection of chicken by both LPAIV and HPAIV induces high levels of IFN in the lung, despite the capacity of the viruses employed to counteract IFN induction in CEF. This represents an important fact for evaluation the function of the NS1 as protein for viral evasion of innate immune responses. As expected, the ability of HPAIV to spread to lymphoid tissue results in much higher systemic IFN responses. Highly virulent H5N1, which cause peracute mortality, induced high levels of IFN. Unfortunately, to our knowledge no comparable data is available for chicken, making it difficult to classify the observed IFN-α response. Nevertheless, with pigs infected with swine influenza only up to 500U IFN-α/mL serum and up to around 10 000 U/g in lung tissue, were found [4]. On the other hand, pigs infected with fatal classical swine fever can have up to 6000 U/mL serum [26], and the highest levels of IFN-α in humans were found with fatal Argentine hemorrhagic fever reaching up to 64 000 U/mL [27]. In both cases of these viral hemorrhagic fevers, case fatally correlated with the levels of IFN-α levis [27-29]. In this context it is also interesting to note that H5N1 can cause coagulopathy with possible dysfunction of endothelial cells and macrophages [30], which are also characteristics of viral hemorrhagic fevers associated with cytokine storm and often high systemic IFN-α levels [29]. Consequently, also based on the potent effect of IFN on the immune system and many physiological processes, it could be speculated that such high systemic IFN responses are detrimental for the host.

It is also noteworthy that the data from the Vac-Y/HA infected group indicate that IFN is not effective in controlling the virus in chicken. In fact, at 3 and 4 days pi, when the animals were euthanized in a moribund state, the viral loads in the lung were still high (10^5^-10^6 ^RNA copies/g; data not shown), although these animals had around 10^3 ^U IFN/mL plasma at this time.

Based on our in vitro results with splenocytes showing IFN responses to viruses as well as inactivated virus we speculate that IFN responses could be of leukocyte origin, possibly from a pDC-like population. The observation that chemically inactivated virus can still activate splenocytes relate to the known biology of pDC in mammals which can respond to inactivate influenza virion. Clearly, future studies are required to identify the cellular source of IFN in chicken.

Considering the correlation of IFN levels with viral loads in lung, plasma and spleen as well as the observation that in vitro splenocyte responses did not differ between the viruses, the high replication speed of the Yama virus may represent may represent one of the key factor determining these responses. In fact this virus has been previously demonstrated to possess a particular rapid and high replication in experimentally infected chicken [19]. Such characteristic can result from a particular efficient polymerase activity [31]. On the other hand, our study also demonstrate the important contributions mediated by HP HA, such as mediated by HP HA, such as the multibasic HA cleavage site cleaved by ubiquitous proteases enabling multiorgan distribution including lymphoid tissue such as the spleen. Furthermore, the HA is known to have an important influence on cellular tropism [32]. Considering the presence of IFN-producing leukocytes therein, this biological property of HPAIV will have an important impact on systemic IFN responses in chicken.

Future studies with IFN receptor knock-out chicken would be required to address the role of systemic IFN responses found with Yama for disease pathogenesis during H5N1 infection of chicken.

## Competing interests

The authors declare that they have no competing interests.

## Authors' contributions

Conceived and designed the experiments: AS, NR, ML, HM. Performed the experiments: HM, ML, SP, MO,LG, AS, NR. Analyzed the data: HM, ML, SP, LG, AS. Wrote the paper: AS, HM, ML. HM and ML equally. All authors read and approved the final manuscript.
